# Assessing Potential Circadian, Diurnal, and Ultradian Variations in Skin Biophysical Properties

**DOI:** 10.7759/cureus.17665

**Published:** 2021-09-02

**Authors:** Harvey N Mayrovitz, Trixie Berthin

**Affiliations:** 1 Medical Education, Nova Southeastern University Dr. Kiran C. Patel College of Allopathic Medicine, Fort Lauderdale, USA; 2 Osteopathic Medicine, Marian University College of Osteopathic Medicine, Indianapolis, USA

**Keywords:** tissue dielectric constant, skin ph, skin blood flow, skin thickness, sebum secretion, transepidermal water loss, tewl

## Abstract

A variety of skin measurements are routinely made in various clinical and research settings to evaluate the skin’s biophysical properties for diagnostic and research purposes. Such measurements include transepidermal water loss (TEWL), skin pH, sebum, skin blood flow (SBF), and tissue dielectric constant (TDC) as a measure of skin water. Given the various reported circadian, diurnal, and possible ultradian and other temporal variations in skin physiological processes, it is of value to have clarity as to possible temporal variations in skin’s biophysical properties associated with such processes. It was thus the purpose of this investigation to review and detail key elements of what is currently known regarding such variations and to provide a characterization that will permit informed judgments as to the sensitivity of the timing of measurements to optimize measurement reproducibility.

Understanding these variations and their possible oscillatory effects on skin biophysical properties may aid physicians in providing optimal treatment timing for dermatological conditions and offer researchers insight into optimal measurement timing. The major findings of the present investigation that systematically searched multiple databases and critically examined pertinent findings, revealed that of the several skin parameters reviewed, which included TEWL, pH, sebum, SBF, TDC, and thickness, each had at least one study describing a statistically significant within-a-day temporal change. The magnitude of these changes varied and may be large enough to be seriously considered when assessing these parameters in clinical and research settings. However, inconsistencies in reported temporal variations suggest that further systematic research is well warranted especially with respect to temporal within-a-day and day-to-day variabilities of TEWL, TDC, and mechanical properties. At present, the impact of this type of confounding variability on reported values for skin biophysical parameters is unclear and worthy of further clarification.

## Introduction and background

Circadian rhythms refer to the body’s endogenous 24-hour physiologic, metabolic, and behavioral rhythms [1]. Circadian rhythms are influenced by light and the environment and are controlled by a central regulator located in the suprachiasmatic nucleus (SCN) of the anterior hypothalamus [1]. Different types of skin cells contain distinct circadian clock machinery that autonomously drives its particular skin function [2]. Regulated circadian activities allow the skin to adapt daily functions to variations in environmental conditions [2]. It has been suggested that 24-hour rhythmicity is best studied over a continuous time span of at least 48 hours [3]. In contrast to the 24-hour cycle of circadian rhythms, an ultradian rhythm can achieve more than one cycle within a 24-hour time span. A diurnal rhythm may be defined as a rhythm that happens, occurs, or is active during the daytime and is synced by day and night. Examples of physiological circadian rhythms previously studied include the sleep/wake cycle [4], blood pressure [5], heart rate and its variability [6], and cortisol secretion [7]. Under controlled environmental conditions, rhythmic variations of skin surface parameters have also been reported [8]. The present study deals with temporal variations and potential rhythms associated with skin biophysical properties.

The skin, the largest organ, functions as a barrier between the internal and external environment of the human body. The three major layers - epidermis, dermis, and hypodermis work together to protect the host by their wide array of functions: physical permeability barrier, ultraviolet protection, thermoregulation, antimicrobial activity, homeostasis, regeneration, and DNA repair. Skin is complex, containing multiple cell types and structures [9]. The epidermis and outer stratum corneum play the largest role in the skin’s biophysical barrier. The epidermal layer shows rhythmic physiological responses to daily environmental variation (e.g., DNA repair) [10]. Individual and environmental factors play a role in the condition of these two layers. 

Some analyses of potential circadian or diurnal rhythms assume that the change pattern is close to a cosine function [11]. This can be useful since the cosine function is a well-described mathematical function and various software programs are available to do semiautomatic analyses based on the cosine function. When used for rhythm analysis, this method is called cosinor analysis, which quantifies 24-hour and other cycles by examining the degree of “fit” between measured data and a user-defined model [12]. Using such cosinor methods, a variable called the acrophase is identified as the time of day where the circadian cycle obtains its maximum [13]. The acrophase defines the peak time of a rhythm and is usually given with narrow and symmetrical confidence limits. However, if the experimental data are far from a cosine function or the acrophase confidence limits are wide and asymmetric, the applicability of this method is limited, especially if the sample size is small [11]. In fact, it has been suggested it not be used if acrophase confidence limits are larger than ±2 hours and have an asymmetrical distribution [14]. However, there are a variety of analysis methods that may be employed to extract potential rhythmicities of time-series including spectral analysis [15] and analysis of variance [16]. More in-depth discussion of the cosinor method is available for the interested reader [17].

Many skin measurements are routinely made in various clinical and research settings to evaluate the skin’s biophysical properties for diagnostic and research purposes. Such measurements include skin transepidermal water loss (TEWL) [18], pH [19], sebum excretion [20], blood flow [21], tissue dielectric constant (TDC) [22], and other skin electrical and mechanical properties [23,24]. Some of these skin properties have been reported to display oscillatory patterns with features that depend on the skin region measured, and the time in which they are measured [8,25,26]. However, there has not been a recent systematic review of such temporal variations. Consolidation of this information is likely to be of value in both clinical and research settings since the timing of such skin measurements may be an unknown confounding parameter if significant temporal variations are present. 

## Review

The purpose of this study was to investigate and summarize the evidence for temporal variations in skin properties including those that may be characterized as circadian, ultradian, or diurnal. 

Methods

A systematic literature search was done on peer-reviewed journal articles written in English using the following electronic databases: Biomedical Reference Collection: Comprehensive Edition, PubMed, Cumulative Index of Nursing and Allied Health Literature (CIABHL) Complete, Web of Science, and Excerpta Medica database (EMBASE). The search was completed on August 1, 2021, and used the following search terms in the article title: “circadian and skin”, “diurnal and skin”, “ultradian and skin”. There was no restriction on publication year. After removing duplicates, a total of 160 articles had data on human subjects and were evaluated for relevancy and the most relevant were included in this review.

Skin pH and its variability

Normally, the skin’s surface has an acidic pH that helps control resident skin microflora. Its value and potential variability were assessed in a large study in which pH was measured on the volar forearm of 330 subjects from four countries: Netherlands, Germany, Philippines, and Spain [27]. The pH was measured between 10:00 and 12:00 hours and again during the same interval 24 hours later. Subjects refrained from any contact with water or cosmetic products between the first and last assessment. Initial and final pH values were reported as 5.12 ± 0.56 vs. 4.93 ± 0.45 respectively. The authors interpreted these results as evidence that when extraneous factors are removed, the 24-hour pH value is close to a ‘natural’ skin surface pH value. Further analysis of the pattern of change led the authors to estimate that the natural average skin pH is 4.74 [27]. Although these authors don’t report values separately for men and women, they state that men’s pH values were lower than for women. These findings add little to understanding pH temporal variability but do help define its expected average value and the need to control for confounding variables.

Temporal variability in pH was further studied in a group of 20 young healthy males in whom axillary skin pH values were measured [28]. The premise of this study was that skin surface pH is influenced by eccrine sweat gland activity, which itself varies during the day. In this study, subjects refrained from using underarm antiperspirant for 17 days and then they removed underarm hair 24-48 hours prior to testing. During the test, they were thermally stressed in a climatic chamber set at 40^o^C and 40% relative humidity for 40 minutes between 10:40 and 11:40 hours and then again between 15:40 and 16:40 hours. Before measuring, the axillary region was wiped with distilled water and blotted dry with a tissue to remove excess sweat. A small but statistically significant difference (p=0.023) was reported in pH between morning pH 5.87 ± 0.23 and afternoon 5.49 ± 0.23. The authors concluded that this demonstrates a skin pH diurnal rhythm. However, recalculation of their primary data by the present authors indicates the same means as previously reported, but with wider standard deviations being 0.51 and 0.52 respectively. Further, despite the wider variance, paired analysis indicates a highly significant difference between morning and afternoon pH values (p<0.001). Although the explanation for the discrepancy in calculations is unknown, it appears that the pH differential reported is valid. A possible explanation may be deduced by considering a diurnal increase in eccrine sweat rate that causes less reabsorption of bicarbonate rendering surface sweat to have a reduced pH as these authors observed. 

Other skin sites (forehead and volar forearm) have been studied in search of temporal or rhythmic variations. Le Fur et al. [8] assessed skin pH and other skin parameters at contiguous four-hour intervals in a group of eight Caucasian young women over a 48-hour time frame. The women were maintained in well-controlled environmental conditions and standardized eating patterns throughout the 48-hour study. The diurnal activity was synchronized by lights on at 08:00 hours (±1 hour) and lights off at midnight (±1 hour). Results indicated no significant day-to-day differences detected, with forehead values ranging from 4.9 to 6.3 and forearm values ranging from 5.4 to 6.6 with no cosinor rhythm being detected at either site. However, when expressed as a percentage of the 24-hour mean, a clear nocturnal dip in pH was detected on the forehead that occurred at 04:00 hours. Analysis of variance showed an overall time-dependent significance (p<0.03) with a forehead nocturnal dip of 5% of the 24-hour mean.

In contrast to these findings, a cosinor analysis did detect a diurnal forearm pH peak in a study in which skin pH, TEWL, and skin temperature were measured in 16 healthy volunteers at forehead, forearm, upper back, and both shins. Subjects (23 to 57 years, nine men and seven women) were measured at two-hour intervals over a 24-hour span [26]. All subjects followed similar diurnal activity patterns and standardized climatic and environmental temperatures while at the testing laboratory. Circadian rhythms based on cosinor analysis were detected for forearm (p=0.002) and shin (p=0.04), but not for other sites. The forearm pH acrophase was recorded at 13:40 hours, and the shin acrophase at 11:00 hours. Peak to trough differences was statistically significant (p<0.05) and ratios were reported as follows: forehead 5.29/4.93, forearm 5.44/4.87, upper back 5.5/5.14, shin 5.5/4.8. These findings are in contrast to results for axillary skin pH [28], which had the highest pH in the morning and lowest pH in the afternoon.

Other evidence of pH variations was reported based on data obtained from 12 healthy, young (23-39 years) Middle Eastern persons. They were evaluated after 30-minute stabilization in a room with a climate-controlled temperature of 22^o^C and relative humidity of 30-40%. TEWL, pH, and sebum excretion of their mid-forearm was measured at 08:00, 12:00, and 16:00 hours [29]. The pH values at 08:00 hours of 5.72 ± 0.48 were reported to be higher than 5.33 ± 0.55, which was measured at 16:00 hours (p=0.001). These results suggest that skin pH is less acidic in the early morning compared to later in the day. However, the other parameters measured were reported to not show significant variation.

It is noteworthy that it is not uncommon for different rhythms to be reported for the same parameter in different studies. Reasons for these inconsistencies may be related to differences in methodology. As an example, one group collected results in a 24-hour time span, resulting from two sessions of 12 hours, one week apart [11]. Time-related changes in the 24-hour scale were reconstructed rather than measured. According to a critical view, 24-hour rhythmicity is best studied over a continuous time span of at least 48 hours [3] although the rationale for this was not explained. Thus, there is controversy on the proper methodology and approach in this field.

Transepidermal water loss and its variability

TEWL refers to the amount of water vapor lost through the skin [30]. In the absence of sweat gland activity, it reflects stratum corneum barrier function, and TEWL is used to characterize the efficacy of this barrier function. Measurement requires placing a device in contact with skin that captures escaping water vapor and expresses this water loss in grams per square meter per hour (g/m2/h). 

TEWL temporal variability has been studied by several groups targeting multiple anatomical skin areas. TEWL measured on cheeks and forearm of eight young (24 ± 3 years) Caucasian women at contiguous four-hour intervals over 48-hours were reported to show significant rhythms via cosinor analysis, with peaks in TEWL on cheek at 08:00 and 16:00 hours and minimum values at 20:00 and 24:00 hours [8]. The 24-hour mean TEWL across subjects ranged from 9.9 to 19.2 g/m2/h. On volar forearm, TEWL peaks were also observed at 08:00 and 16:00 hours, with troughs at 12:00 and 24:00 hours. The forearm 24-hour mean TEWL ranged from 5.9 to 10.4 g/m^2^/h. In addition to the 24-hr rhythm component, ultradian rhythms with periods of eight and 12 hours were reported for TEWL of face and forearm. The circadian maximum amplitude of facial skin was higher than the ultradian amplitude, and vice versa for forearm. These findings suggest that TEWL has a circadian rhythm on face and an ultradian rhythm on forearm. However, In direct contrast to these findings, TEWL measurements on forehead, forearm, and upper back were reported to have higher TEWL values in the evening (20:00 hours) and minimum values in the morning (08:00-10:00 hours) [26]. In this study conducted on 16 healthy subjects (nine male), peak to trough differences were reported as 36/9 for forehead, 16/9 for forearm, and 32/12 for upper back. All differences were reported as statistically significant (p<0.05). The higher TEWL in the evening suggests decreased epidermal barrier function with a possible increase in topical drug permeability. Cosinor analysis of the circadian changes of TEWL in this study compared to others [8] show considerably different acrophases. For forehead one was 11:30 hours and the other 06:00 hours. For forearm it was 24:00 vs. 18:00 hours. These differences again point out the uncertainty in the precise nature of the temporal variability in TEWL.

A few other small studies have investigated TEWL variations. In one, TEWL was measured in six subjects at 09:00, 14:00, and 19:00 hours at unreported anatomical sites [31]. In five of these subjects, higher TEWL values were obtained in the afternoon and evening and compared to the morning. The authors reported a diurnal variation in TEWL. In another small study, TEWL was measured in eight Caucasian men and nine Caucasian women at multiple forearm sites [18]. Measurements were done at two-hour intervals from 09:00 to 17:00 hours in a climatic chamber maintained at 40% relative humidity and 21^o^C. Subjects were placed in the chamber for a minimum of 30 minutes before each measurement. Between measurements, subjects were confined to a resting room and instructed not to exercise. Measurements indicated TEWL decreased by 9%, from 5.07 ± 0.13 g/m2/h at 09:00 hours to 4.61 ± 0.09 g/m2/h at 17:00 hours (p<0.05). These small studies, although not detecting rhythms, emphasize the possible time-dependent variability of TEWL. Contrastingly, in a very well-controlled study in which forearm TEWL was measured in 16 young women over 48-hours at four-hour intervals, a clear temporal variation in TEWL was shown with a maximum at 04:00 hours and a minimum at 14:00 hours [32]. Examining these results indicates a max/min ratio of about 2.2 for women not using oral contraceptives and 2.0 for women using oral contraceptives.

Skin blood flow variability

Two major functions served by SBF are temperature regulation and skin nutrition. Although the nutritional component under resting conditions is relatively constant, the component that serves the thermoregulatory function varies with environmental and skin temperature. Thus, in any evaluation of possible circadian or other rhythms, it is important to maintain temperatures and conditions relatively constant. This is a demanding requirement and evaluation of reported findings must take this into consideration. One method widely used to obtain an index of SBF is that of laser Doppler flowmetry (LDF) in which the Doppler shift in laser light penetrating the skin and then reflected is used to measure the flux of moving red blood cells in the skin’s small blood vessels. This can be done using a sensor applied directly to the skin [33,34] or by a laser beam that scans the skin area known as laser Doppler imaging (LDI) [35]. Using the LDI method, SBF was measured on the proximal and distal forearm of nine healthy Chinese subjects (four men and five women) once every three hours for a 24-hour period [25]. SBF showed a circadian variation in the proximal and distal forearm (p=0.04, p=0.02, respectively). A deep circadian trough was detected at 08:00 hours, with a peak at 20:00 hours. An ultradian rhythm was detected to be statistically significant only on the distal forearm (p=0.01). Peaks of the 12-hour ultradian variations were between 13:00-14:00 hours and between 01:00-02:00 hours. 

Another attempt to assess circadian variation investigated the SBF response to passive heat stress. This was done in six healthy men at four different times of day (04:00-07:00, 10:00-13:00, 16:00-19:00, and 22:00-01:00 hours) [36]. Each time of day was evaluated on separate days by a laser-Doppler flowmeter placed on the right side of the back and on the volar forearm. After a seated rest period of 15-minutes, heat stress at rest was introduced by immersing the subject’s legs below the knee in 42^o^C water for 45 minutes. Results indicated that maximum SBF failed to show a significant difference among times of the day. A follow-up study done by these investigators compared SBF to the local sweating rate [37]. Results indicated that the increased maximum SBF without sweating was significantly higher at night than in daytime and evening for both body sites (p<0.05). These results suggest a circadian variation in the SBF response before the onset of sweating during passive heat stress. However, the question of whether basal SBF displays a circadian or diurnal variation still remains unanswered. One attempt to deal with this issue was done by monitoring SBF on the finger dorsum of 15 healthy fasting resting men using the LDF method [38]. Measurements were made after 60-minute acclimatization with the men in a supine position. SBF was measured at six times (09:00, 10:30, 12:00, 13:30, 16:30, 17:30 hours) with each measurement lasting two minutes. During the 10-hour experimental interval, the men were allowed to sit up in a reclined position but had to be back in the supine position 15 minutes prior to measurements. After careful analysis of variance, the results failed to demonstrate a diurnal variation in SBF.

Sebum excretion variability

Sebaceous glands are skin appendages that produce sebum through a holocrine mechanism [39]. Sebum excretion governs sebum outflow to the skin surface through these glands [40]. Two parameters may be used to quantify sebum excretion. One is the static or the casual level that refers to the skin surface lipid amount of a non-protected area neither wiped nor washed. The other is dynamic that refers to sebum excretion rate (SER), which is skin surface lipid flow of a previously cleaned area [39]. Sebum excretion can be measured through numerous methods. The gravimetric method uses an absorbent paper that collects the sebum. Another method uses Sebutape® (CuDerm Corp, Dallas, US), a lipid-absorbent transparent film that tightly applies to the skin surface and collects surface sebum. Skin pores filled with sebum will block the light and form spots on the film thereby allowing assessment of skin surface sebum. This method was used to measure chest and forehead sebum excretion of 12 healthy male volunteers, separated into two groups of six studied four to five days apart with their measurements pooled [39]. Subjects were housed in a test facility for 31 consecutive hours and synchronized with a diurnal activity from 07:00 to 23:00 hours and nocturnal rest. After swabbing test sites with alcohol, a Sebutape patch was applied for one hour, then removed and placed on black paper to be photographed. Image analysis was used to determine the number of active follicles by the number of sebum spots per square centimeter and the total amount of sebum secreted in each one-hour period. This procedure was repeated every two hours throughout the 30-hour test. Results for forehead and chest were characterized by steadily decreasing sebum from 12:00 to 04:00 hours with about a 50% reduction but based on cosinor analysis revealed a circadian pattern on forehead (p<0.001) with a peak value at 13:00 hours. Thus, based on these data, the SER was lowest during the night and peaked in mid-afternoon. Some earlier work has indicated the presence of circadian rhythms in sebum secretion in most persons that peaks in the late morning [41]

Tissue dielectric constant

Skin TDC values largely depend on tissue water content [42]. Thus, such measurements are useful to assess potential hour-to-hour or day-to-day changes in skin properties related to water holding features. Such data is sparse in the literature. However, one study approached this by self-measurements made by 12 young women hourly starting at 08:00 hours and ending at 20:00 hours [43]. TDC was measured bilaterally in triplicate to a skin depth of 2.0-2.5 mm at four sites; face below the eye, mid-cheek, forearm, and calf. TDC values from the face and forearm progressively decreased (p <0.001) from morning to night. Decreases ranged from 11.2 ± 8.3% on the face to 5.6 ± 6.0% on forearm with correlation coefficients ranging from 0.708 to 0.941. Contrastingly, TDC at calf significantly increased (p < 0.001) with a correlation coefficient of -0.864 with a morning-to-night increase of 9.3 ± 10.7%. Day-to-day variability was not assessed in this study but could provide additional information on the repeatability of the findings and further studies using this modality appear warranted. 

Skin thickness variability

Skin thickness and its variability can be determined via high-resolution ultrasound that has been reported to have good repeatability within and across examiners [44] providing a noninvasive method to measure skin thickness [45]. If B-mode ultrasound is used, the echogenic part of the ultrasound image is proportional to tissue water content [46]. This method was used to measure skin thickness at multiple anatomical sites in 20 Japanese men and 20 Japanese women at two time-points, 08:30-10:30 hours and again at 15:30-17:00 hours. Sites were: forehead, cheek, corner of the eye, forearm, upper arm, the flank, thigh, and calf, with measurements done in a room at 23^o^C and relative humidity of 40% [47]. Skin thickness in both sexes significantly decreased in all upper body areas but increased in thigh and calf (p<0.005). Although numeric data was not presented, the present authors estimated the changes from their table 2B as thickness decreases for eye, cheek, and arm of 7%, 10%, and 12%, and an increase of 15% at the calf. This differential diurnal change between the upper and lower body suggests that skin thickness varies diurnally with dermal fluid volumes. This is consistent with the TDC measurements previously reported [43]. However, although this study demonstrated a temporal change in skin thickness, the experimental design was not adequate to detect specific rhythms.

In a follow-up study, 38 healthy Japanese subjects (22 males and 16 females) with wrinkles on their forehead, corner of the eye, and nasolabial grooves were evaluated at the same clock-times and under the same conditions of temperature and humidity as the prior study [48]. The skin in each measurement area was significantly thinner in the afternoon than in the morning (forehead p<0.05, corner of eye p<0.001, nasolabial groove p<0.001) suggesting a skin thickness diurnal effect. Another study by this group focused on determining if the diurnal variation of skin thickness is modulated by aging effects. Such aging effects were characterized as having or not having facial wrinkles (forehead, corner of eye, and cheek). In this study, 130 healthy Japanese females were evaluated under similar conditions but only weak correlations with diurnal changes were found [49]. However, the basic pattern of a diurnal decrease in the upper body (face) skin thickness was also present. Thus, based on these ultrasound studies, gravity-related shifts of dermal fluid from the face to the legs, at least in part, are responsible for the diurnal variation in thickness. Such changes likely affect skin mechanical properties but available literature on this aspect is essentially silent, suggesting an area in need of future research.

## Conclusions

The findings of the present investigation revealed the following major features: of the several parameters reviewed, including skin pH, TEWL, skin blood flow, sebum excretion, tissue dielectric constant, and skin thickness, each had at least one study describing a statistically significant temporal change. The magnitude of these changes varied and, in most cases, may be large enough to be seriously considered and taken-into-account when assessing these parameters in clinical and research settings. However, various inconsistencies in reported temporal variations suggest that the issue of significant circadian, diurnal, or ultradian variations in the biophysical features is as yet uncertain. This indicates that further systematic research is well warranted especially with respect to TEWL, TDC, and skin mechanical property temporal variabilities within-a-day and for day-to-day variations. At present, the impact of this type of confounding variability on reported values of skin biophysical parameters is unclear and worthy of further research. The present findings also have implications for cosmeceutical testing and development and other areas of skin measurements and suggest that when skin measurements are reported, the time of day of these measurements should also be reported.
